# Establishing a Cone Beam CT-Guided Bronchoscopy Program: A Stepwise Guide for Interventional Pulmonologists

**DOI:** 10.3390/diagnostics16111616

**Published:** 2026-05-25

**Authors:** Sammy Onyancha, Naveed Mohamoud Merali, Nishma Elesh Gajjar, Peter Waweru Munyu, Angelique Holland, Gernot Rohde

**Affiliations:** 1 Department of Pulmonology, St. Elisabethen Krankenhaus, Ginnheimer Str. 3, 60487 Frankfurt, Germany; 2 Department of Respiratory Medicine, Avenue Hospital, Nairobi P.O. Box 45280-00100, Kenya; 3 Department of Respiratory Medicine, Universitätsklinikum Marburg, 35043 Marburg, Germany

**Keywords:** cone beam CT, CBCT, navigational bronchoscopy

## Abstract

Cone beam computed tomography (CBCT)-guided bronchoscopy has emerged as a powerful tool in the diagnosis of peripheral lung lesions, offering real-time, image-confirmed biopsy capabilities that enhance precision and diagnostic yield. However, implementation of a CBCT program presents significant logistical and technical challenges. This article presents an experience-based implementation tutorial outlining a stepwise approach to establishing a CBCT-guided bronchoscopy program. The framework is derived from iterative workflow development across more than 300 procedures at our institution, St. Elisabethen Hospital in Frankfurt, Germany, as well as our implementation efforts at Avenue Hospital Parklands in Nairobi, Kenya. Key domains addressed include infrastructure assessment, access strategy, training, procedural logistics, ventilation protocols, case selection, workflow optimization, safety considerations, and business case development. This roadmap aims to support interventional pulmonologists in integrating CBCT into clinical practice, while emphasising the need for local adaptation based on institutional resources and multidisciplinary collaboration.

## 1. Introduction

**Figure 1:**
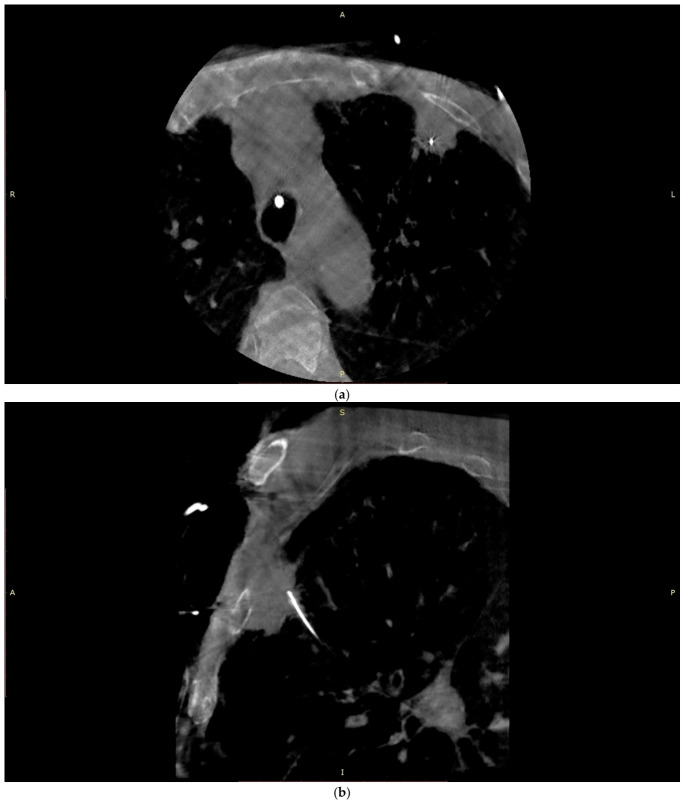

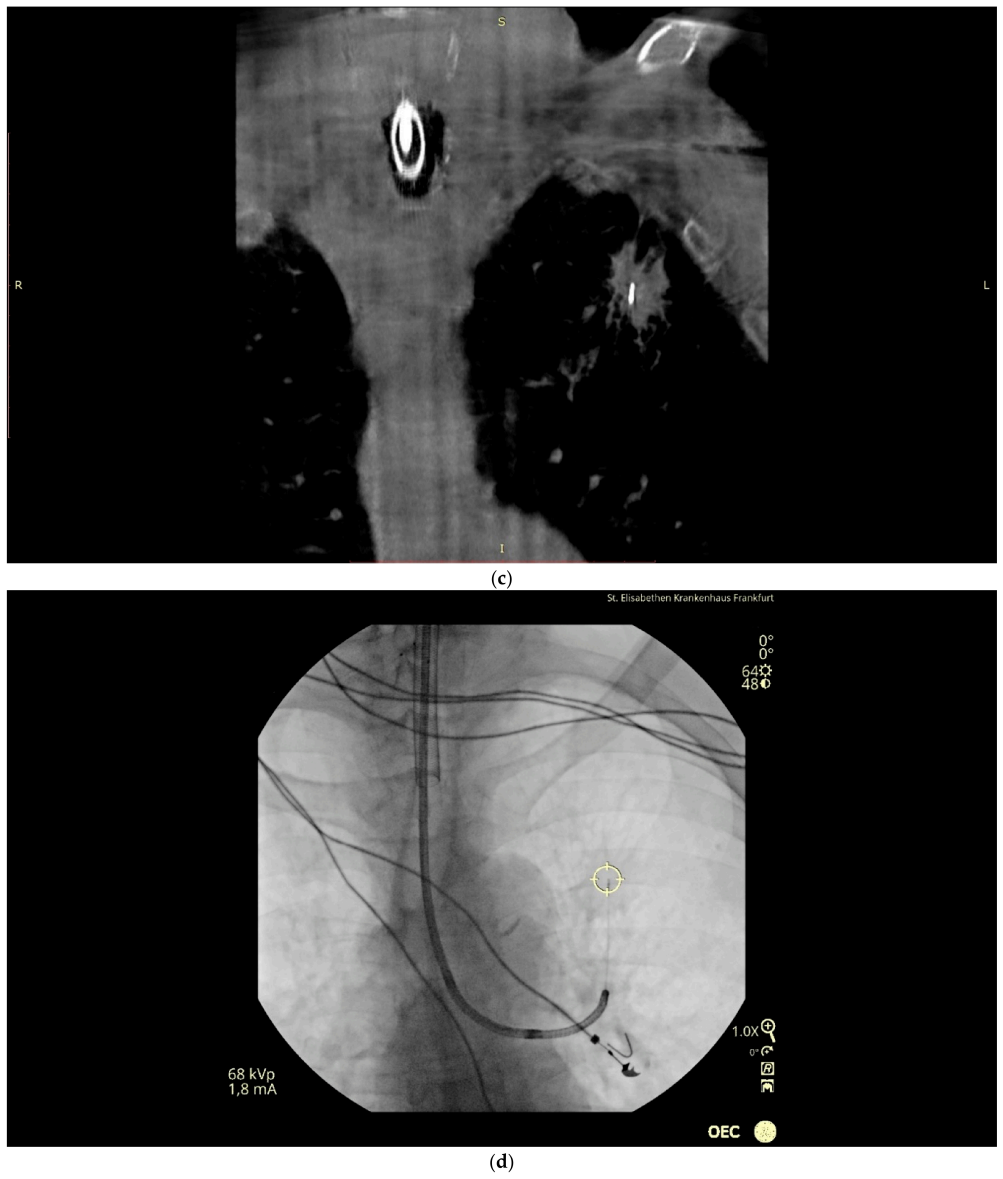
(**a**–**d**): Intraoperative CBCT images showing biopsy tool-in-lesion.

Despite advances in navigational platforms, the diagnostic yield for peripheral lung nodules with bronchoscopy has historically plateaued. CBCT-guided bronchoscopy offers a paradigm shift, allowing for real-time, three-dimensional verification of “Tool-In-Lesion” (TiL) (Figure 1a–d), which presents a critical milestone toward accurate diagnosis and safe future therapeutics [1,2,3].

Numerous studies have focused on diagnostic performance; however, fewer publications address the practical challenges associated with establishing a CBCT program at an institutional level. Implementation requires coordination across departments, adaptation to new imaging workflows, and iterative refinement of procedural logistics. These factors represent a substantial barrier for centres seeking to integrate CBCT into routine bronchoscopic practice.

**Figure 2:**
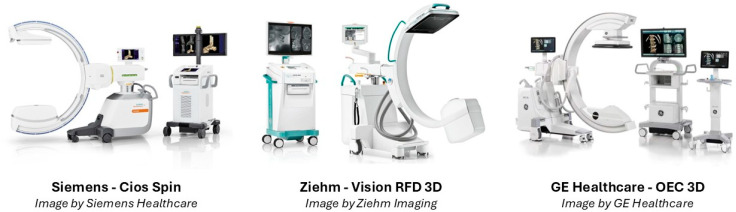
Mobile CBCT scanners.

Although dedicated mobile CBCT units are increasingly being integrated into endoscopy suites at high-volume centres (Figure 2), most institutions initially rely on shared access to existing stationary or mobile CBCT systems located within hybrid operating rooms or interventional radiology environments. For many centres, this shared-access model represents the most practical pathway for early adoption prior to investment in a dedicated endoscopy-based CBCT platform. This tutorial is intended to support the initial implementation of CBCT-guided bronchoscopy through structured utilization of shared operating room CBCT resources, with the longer-term objective of establishing a sustainable and dedicated CBCT-guided bronchoscopy program.

This article is presented as an experience-based implementation tutorial, with recommendations derived from retrospective evaluation of institutional workflow development across more than 300 CBCT-guided bronchoscopies, iterative protocol refinement during program establishment in two centres, and consensus among the authors based on multidisciplinary collaboration. Where available, recommendations are supported by the published literature; however, several elements, particularly workflow design, logistics, and infrastructure adaptation, are based primarily on institutional experience.

## 2. Step-by-Step Guide

### 2.1. Step 1: Infrastructure Audit

The first step to implementing a CBCT program is to understand your institutional infrastructure as well as the politics behind it. You need to first identify whether a CBCT-capable scanner exists, including both fixed room-based systems and mobile CBCT-capable C-arm systems. These are commonly located within interventional radiology (IR), cardiology, or hybrid operating rooms (ORs) for orthopaedic or vascular surgery, while mobile CBCT systems may be shared across multiple procedural areas or stored centrally depending on institutional workflow. After locating the scanner, you need to find out the technical capability of the scanner and judge its compatibility with your procedural needs (e.g., does the scanner have augmented fluoroscopy or what is the image acquisition time) [Table 1]. Then, review scheduling patterns to identify underutilized slots or machines. At this point, determining who oversees scanner allocation and slots is important to know who to contact to get permission for use.

### 2.2. Step 2: Entry Strategy

One of the major early hurdles is gaining initial access to the scanners. Most of the time, even though OR scanners may be underutilized, the other departments will not allow for them to be taken away or shared, as this encroaches on their unrestricted control and use of the scanners. Finding a reason to gain access without disrupting existing departmental workflows is a challenge. Developing a rapport with vascular surgeons or the interventional radiologist may help ease gaining access. A strategic entry point to gain access is a planned investment. Leveraging a potential purchase of a CBCT unit for your endoscopy unit may help gain access without seeming threatening, because the purchase would mean the surgeon’s use of the CBCT scanner would not be reduced due to your presence. Ask for an observership of the CBCT as well a test run of the unit as a “try-before-you-buy” scenario in order to gain more experience before your purchase is done. This would justify your use of the system and your presence without seeming too invasive. Additionally, engage radiologic technologists and scanner staff to understand workflow and politics.

### 2.3. Step 3: Case Preparation

Preparing before the first case involves four key aspects—training, logistics, ventilation, and safety.

**Figure 3:**
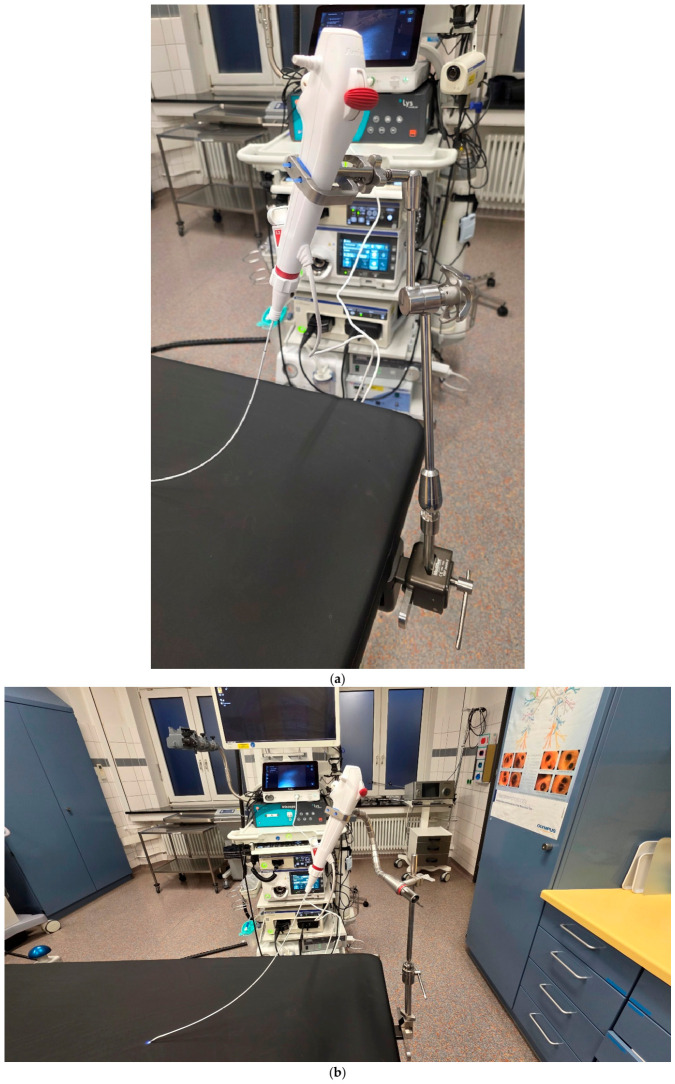

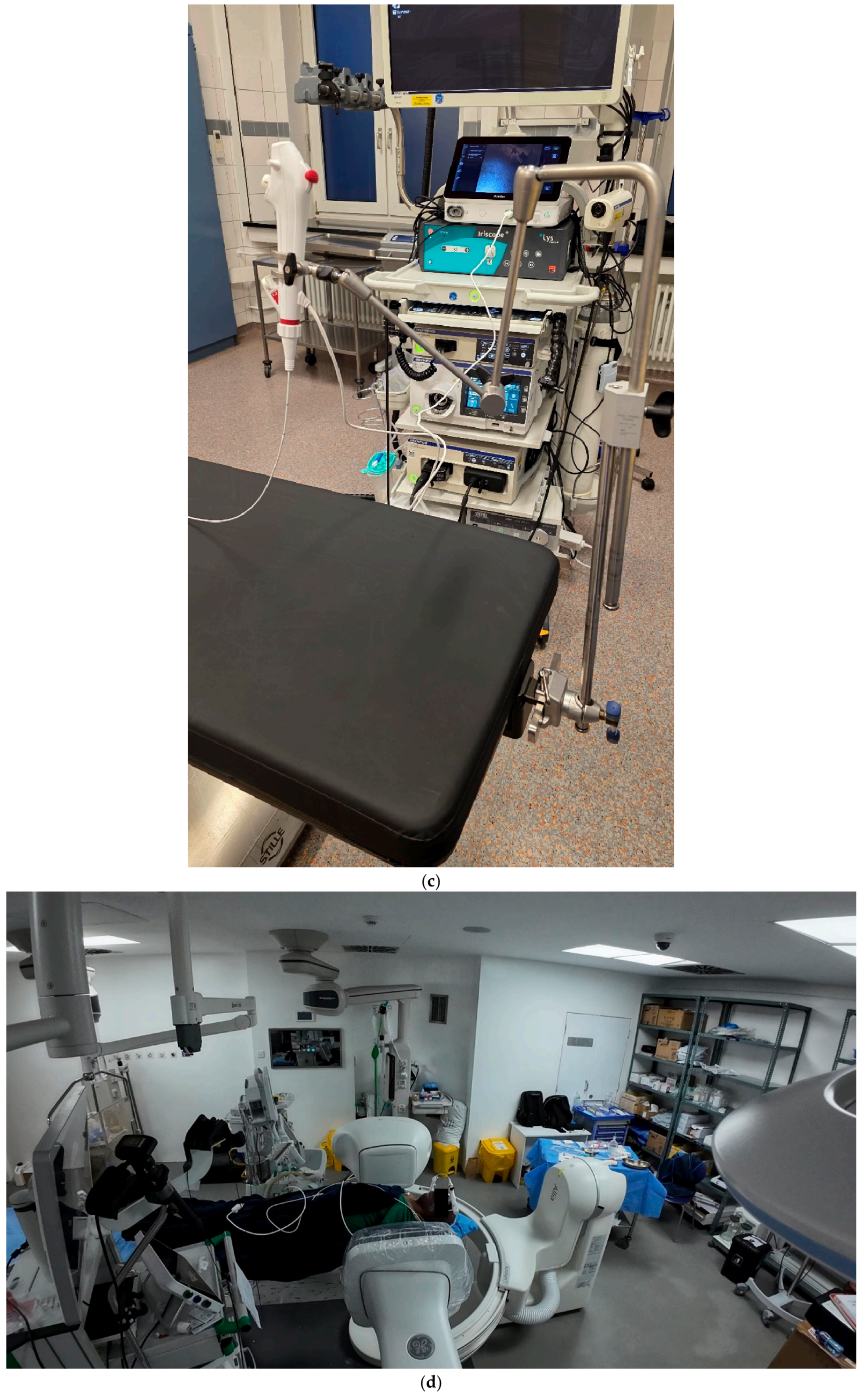

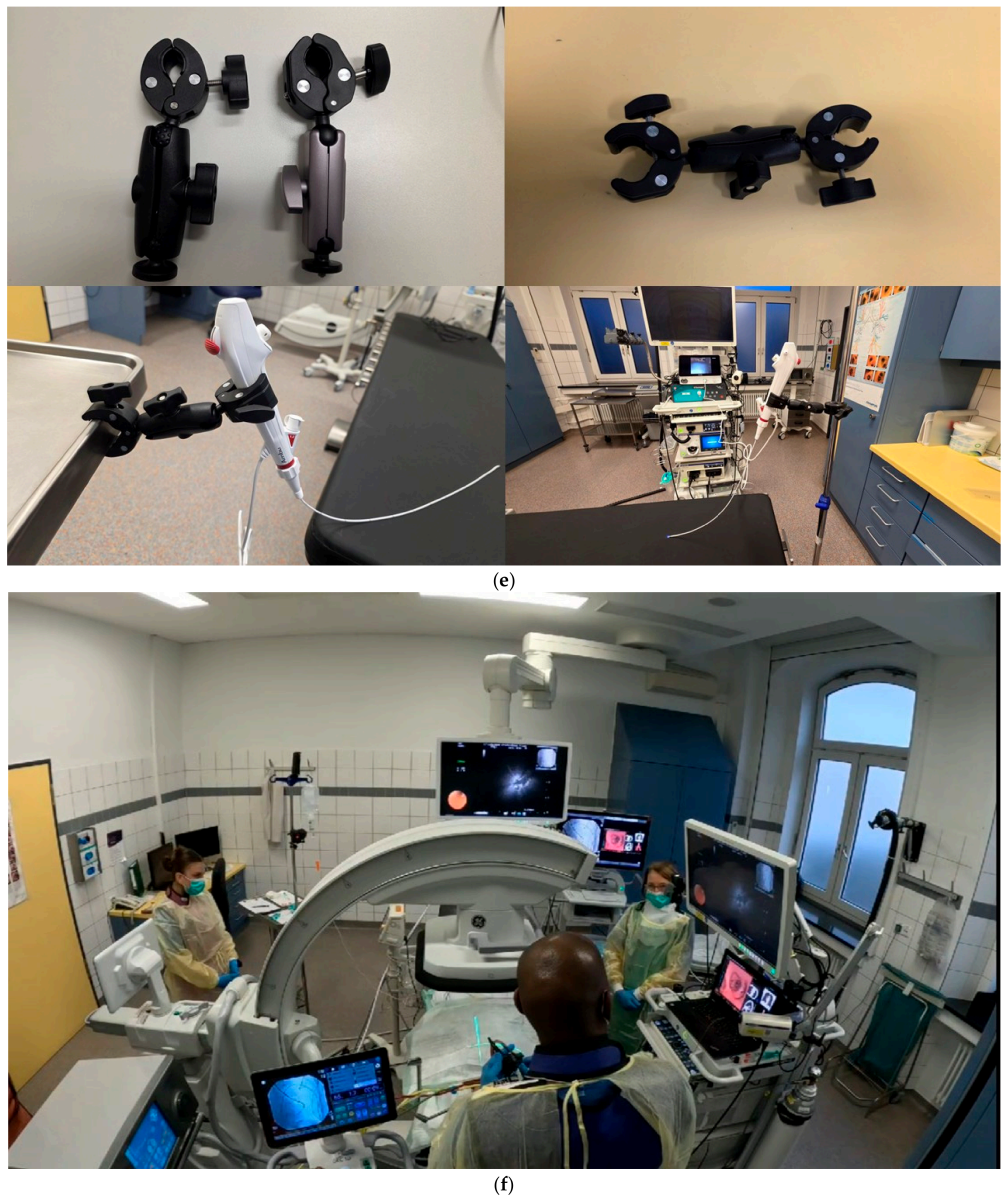
(**a**,**b**): Standard flexible bronchoscope holder/fixation systems. (**c**): Tracheoscope mounting system used as bronchoscope stabilization device. (**d**): Single-use bronchoscope taped to patient during CBCT spin. (**e**): Action camera mounting systems converted to bronchoscope holders to mount bronchoscope to stationary objects. (**f**): Room layout during CBCT interventions.

- Training: Visiting and consulting centres with established CBCT programs can help you understand setup and implementation. The direct exposure to experienced operators can accelerate the learning curve and help anticipate challenges during program implementation. In situations where in-person observerships are not feasible, digital engagement offers a practical alternative. Digital observership remains a budding substitute for in-person shadowing [4]. Several online platforms, as well as some hospitals, allow for online observership of live cases. Similarly, video footage of CBCT biopsies, as well as talks/webinars on the subject, are available online, which can help guide the initial setup.
- Logistics: A thorough understanding of the procedural room layout facilitates smooth initial integration. Careful planning of the bronchoscopy tower position and ancillary equipment, such as the ventilator, is essential to avoid intraprocedural complications and workflow interruptions. A common initial bottleneck is the availability of a bronchoscope holder, as CBCT procedures require the bronchoscope to remain fixed in a stable position while the procedural team exits the room during imaging (Figure 3a,b).

The lack of a dedicated mounting system is one of the main barriers to CBCT implementation. If a standard bronchoscope holder is unavailable initially, several practical alternatives may be considered. During our early Frankfurt cases, a rigid tracheoscope holder was successfully adapted as a mounting device (Figure 3c).

In many settings, standard laparoscopic mounting systems—particularly cystoscope holders—may serve as suitable substitutes; collaboration with the urology department can therefore be advantageous. During the initial implementation phase in Kenya, no holders were available, so single-use bronchoscopes were used, as their low weight allowed them to be securely taped adjacent to the patient while maintaining stability (Figure 3d).

An off-label option includes action-camera bicycle mounts (Figure 3e), which can secure the bronchoscope to a stationary structure; however, precaution is advised with such options, and they should only be used following institutional risk assessment and approval, ensuring compliance with sterility and safety requirements.

Finally, the bronchoscopist’s position relative to the patient and imaging system should be considered early, as it significantly influences procedural ergonomics and overall workflow (Figure 3f).

Hosting a “dry run” with the team to simulate the procedure can help expose workflow gaps and help familiarize support staff with the unique demands of CBCT-guided bronchoscopy.

- Ventilation: Intraprocedural atelectasis can obscure peripheral targets, worsen CT-to-body divergence, and generate false-positive radial-probe endobronchial ultrasound images, thereby reducing diagnostic yield [5,6,7,8,9]. For this reason, ventilation and patient positioning should be planned before the first case and aligned with the anaesthesia team. A pre-procedural discussion with the anaesthesiologist is essential to define the most feasible institutional strategy, as the optimal approach may vary according to local equipment, anaesthetic practice, lesion location, and patient characteristics. Key decisions include the ventilation mode, such as high-frequency jet ventilation versus pressure-controlled ventilation, and the airway conduit, including rigid bronchoscope, laryngeal mask airway, or endotracheal tube. In many cases, minimizing target motion is as important as preventing atelectasis. Motion-reduction strategies may include prolonged inspiratory phases during CBCT acquisition through adjustment of the inspiratory-to-expiratory ratio, lower tidal volumes, controlled breath-holds, or high-frequency jet ventilation to limit diaphragmatic excursion. Atelectasis prevention should be addressed with tailored tidal volume and positive end-expiratory pressure settings, together with recruitment manoeuvres when appropriate. Patient positioning should also be considered, as lateral decubitus positioning has been shown to be superior for preventing atelectasis in dependent-zone nodules [10,11]. Ultimately, the aim is to develop a structured, locally reproducible ventilation workflow that minimizes imaging artefacts and supports accurate tool-in-lesion confirmation. This protocol should be written, rehearsed during dry runs, and periodically reviewed so that it can be applied consistently across anaesthesia teams.
- Safety: CBCT implementation requires adherence to radiation protection and control standards. Staff exposure is minimized by using room-exit protocols during CBCT acquisition. However, the use of CBCT-guided imaging also requires compliance with local regulatory and credentialing requirements related to radiation usage and image acquisition. Operators performing CBCT-guided procedures independently should possess the appropriate institutional and national qualifications for the operation of fluoroscopic and CBCT imaging systems. In centres where bronchoscopists do not hold these qualifications, collaboration with or recruitment of a radiologist or appropriately credentialed imaging specialist may be necessary to support safe program implementation and regulatory compliance.

### 2.4. Step 4: Case Selection

Although it may be tempting to demonstrate CBCT capabilities by selecting a <10 mm nodule as the first case, choosing a straightforward and low-risk large lesion is a far more effective strategy. Early adoption should prioritize simplicity over ambitious case selection. Unlike endoscopy suites, where most bronchoscopic procedures are typically performed, operating rooms may not be equally optimized for managing bronchoscopic complications. Recognizing these environmental differences should encourage careful case selection during the early implementation phase. Choosing a low-risk, easily accessible target lesion not only improves procedural efficiency, but also builds trust with anaesthesia teams and operating room staff. Completing a safe, uncomplicated case within approximately 20 min is far more likely to foster future collaboration than a prolonged, complication-prone procedure. The purpose of the first case is not to fully demonstrate the diagnostic potential of CBCT, but to identify workflow limitations, refine logistics, and establish credibility. Early experiences shape perception; a reputation for safety and efficiency will accelerate your integration, whereas early complications may significantly hinder future access to the operating room environment.

### 2.5. Step 5: Team Building

Building the team for the first case is essential. Ideally, you should invite a co-operator with prior CBCT experience to assist. If a co-operator is not available, then an experienced radiology technologist or even technical application specialists would suffice. Most companies are willing to send an applications manager to accompany the initial cases upon request. Their presence can be particularly helpful for troubleshooting should technical issues arise during the procedure. Take advantage of this support during the early phase to facilitate a smoother and more confident implementation of your first cases.

### 2.6. Step 6: Workflow Familiarization

Reviewing the first few cases with an experienced CBCT user can help identify pitfalls of each case. Analyse the procedural times and where the cases could have been streamlined. Regular multidisciplinary meetings can help identify workflow inefficiencies, refine protocols, and foster a shared understanding of procedural goals.

### 2.7. Step 7: Scope Expansion

After performing the first few easy targets, you should have gained the trust and the confidence of the OR team. Having no complications initially makes it more likely that you can receive a fixed OR slot that you can slowly expand on after. Now you can build by expanding to more challenging lesions. However, expansion should still be guided by structured case selection rather than rapid escalation. Factors such as lesion size, location, anticipated navigation difficulty, and patient comorbidity should be always considered during expansion phase. Rather than prioritizing rapid growth, prioritize incremental scaling that preserves procedural safety and team cohesion. This stepwise expansion allows centres to transition from early feasibility toward a sustainable clinical service capable of supporting a broader referral base.

### 2.8. Step 8: Optimization

Once the initial implementation and expansion phases are complete and a stable workflow has been established, the focus should shift toward systematic optimization of procedural efficiency. A practical starting point is the reduction of non-essential procedural steps; streamlining anaesthesia induction, minimizing equipment repositioning, and standardizing pre-procedural preparation can significantly decrease overall case duration. Establishing a fixed room setup, including predefined positioning of the bronchoscopy tower, CBCT gantry, ventilator, and fluoroscopy monitors, reduces variability between cases and improves team confidence. Creating a standardized checklist for equipment and imaging parameters can further enhance consistency and limit delays caused by troubleshooting during the procedure.

Optimization of imaging strategy represents another key element. During early adoption, you may rely on multiple CBCT spins to confirm tool position; however, as experience increases, more selective use of imaging should be encouraged. Identifying when additional scans did not alter clinical decision-making in prior cases can help define a more efficient imaging algorithm. Collaboration with radiology technologists is particularly valuable in this phase, as adjustments in acquisition protocols, field-of-view selection, or augmented fluoroscopy settings may reduce both radiation exposure and procedural time.

Ventilation workflows should also undergo iterative refinement. Close communication with anaesthesia teams allows for the development of a reproducible imaging protocol, including standardized breath-hold strategies or jet ventilation settings tailored to the institutional environment. Establishing clear verbal cues before CBCT spins and defining roles during imaging, such as who confirms bronchoscope stability or manages ventilation pauses, can significantly reduce delays and prevent motion artefacts.

Over time, these measures allow the transition from an exploratory workflow to a reproducible, high-throughput program capable of accommodating increasing procedural volume.

### 2.9. Step 9: Business Case Development

Presenting clinical data from cases performed before and after the implementation of CBCT can provide strong support when proposing a new program to hospital management. Demonstrating improvements in diagnostic yield may strengthen the business case and facilitate strategic planning for system integration.

At our centre in Frankfurt, we conducted a retrospective service evaluation of navigational bronchoscopy procedures conducted between January 2023 and December 2024 prior to the launch of the CBCT program as part of the business plan. Direct costs included consumables (e.g., staplers), operating room (OR) time, and pathology services. OR time was calculated using institutional standard cost-per-minute estimates. Indirect costs such as inter-facility specimen transfer were included where applicable. All costs are reported in Euros (EUR) based on 2024 institutional pricing. From the 720 procedures that were performed, 590 achieved a definitive diagnosis on the first attempt, corresponding to a diagnostic yield of 82%. Repeat bronchoscopy was done in 40 of the remaining 130 initially non-diagnostic cases, resulting in a malignant diagnosis in 35 patients.

For the 95 cases with non-diagnostic results, surgical management typically involved an initial wedge resection with intraoperative frozen section analysis to confirm malignancy before proceeding to definitive anatomical segmental resection. The average waiting time for frozen section results was 80 min. Stapler costs for wedge resections averaged approximately EUR 600 per case, with an average of three cartridges used at EUR 200 each. In addition, frozen section analysis required specimen transport to a neighbouring clinic. Overall, non-diagnostic cases resulted in approximately EUR 59,000 in additional costs, and required 126 more hours of operating room time compared with cases in which a definitive diagnosis allowed upfront surgical resection. This cost analysis supported the investment in CBCT by demonstrating that improved diagnostic yield in navigational bronchoscopy can reduce the need for frozen sections and free operating room capacity for other procedures [Table 2].

Use your clinical outcomes data and cost analysis to advocate for investment in a dedicated CBCT platform within the endoscopy suite, as this not only enables expansion of procedural integration, but also reduces dependence on operating room availability.

### 2.10. Step 10: Leverage Results

Finally, begin promoting CBCT capabilities internally and to referring clinicians, ensuring capacity can meet demand. Hosting institutional case conferences to present CBCT-guided bronchoscopy outcomes can help further expand reach. Highlight difficult cases where CBCT made a decisive impact, particularly in small or apically located nodules, and use clinical images or 3D reconstructions to illustrate value. Additionally, promote externally through CME talks, visiting observerships, or publications and, where possible, collaborate with other departments to integrate CBCT into their workflows (e.g., thoracic surgery or oncology planning).

## 3. Discussion

**Figure 4:**
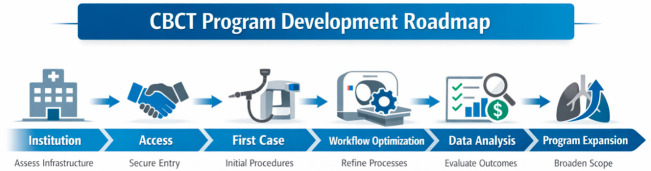
Overview of CBCT program development roadmap.

Establishing a CBCT program is a resource-intensive but transformative endeavour. Success requires careful planning, cross-specialty collaboration, and a mindset shift from traditional navigation to image-guided precision. This experience-based implementation framework highlights practical considerations encountered during program development across different healthcare environments (Figure 4) [Table 3].

While institutional variability necessitates locally adapted solutions, structured preparation, progressive optimization, and continuous performance assessment may support safe and sustainable integration of CBCT into bronchoscopic practice.

Beyond implementation logistics, increasing evidence supports the clinical value of CBCT as a central component of advanced bronchoscopy programs. Meta-analyses and contemporary clinical studies have demonstrated that real-time imaging confirmation of tool-in-lesion is one of the major determinants of diagnostic success during peripheral bronchoscopy [2,3]. Although robotic-assisted bronchoscopy (RAB) has expanded the technical capabilities of navigation platforms, recent reports evaluating RAB combined with CBCT suggest that the incremental benefit is largely derived from CBCT-based imaging confirmation rather than navigation technology alone [12,13]. Concurrent CBCT allows immediate correction of CT-to-body divergence, confirmation of biopsy tool positioning, and optimization of sampling strategy irrespective of the navigation platform used [14].

From a program-development perspective, these findings have important practical implications. Establishing CBCT capability may represent the most impactful initial investment for centres seeking to develop an advanced diagnostic bronchoscopy service, as it provides image-guided verification that can enhance procedural confidence and diagnostic accuracy across conventional electromagnetic, shape-sensing, ultrathin, and robotic bronchoscopic systems. In contrast, robotic platforms may be viewed as complementary technologies that can subsequently expand procedural reach and stability once a mature CBCT-guided workflow has been established. This staged approach may be particularly relevant for centres with limited financial or infrastructural resources, where prioritizing CBCT integration first may offer broader immediate clinical benefit and facilitate gradual program expansion over time.

The implementation of CBCT-guided bronchoscopy also requires recognition that procedural success depends not only on technology acquisition, but also on institutional adaptation. Ventilation protocols, imaging workflows, multidisciplinary communication, and procedural standardization are equally critical determinants of program sustainability [Table 4].

As procedural experience increases, optimization strategies focused on workflow efficiency, radiation reduction, and selective imaging utilization become increasingly important to maintain scalability while preserving procedural quality and safety.

### Limitations

Despite its advantages, CBCT-guided bronchoscopy presents several barriers to implementation. These include limited scanner availability, dependence on multidisciplinary coordination, procedural learning curve, radiation exposure, and cost considerations. Additionally, centres with lower procedural volumes may face challenges in maintaining efficiency and justifying investment. These factors should be carefully considered when planning program adoption.

Additionally, our proposed framework is based on experience from a high-volume centre and one additional site, which may limit generalisability. Institutional variability in resources and expertise may influence applicability. The framework should therefore be interpreted as pragmatic guidance requiring local adaptation.

## 4. Conclusions

As imaging technologies continue to evolve, CBCT-guided bronchoscopy is likely to play an increasingly central role in the management of peripheral lung lesions. The ability to combine precise real-time imaging with advanced bronchoscopic navigation creates a platform for increasingly accurate, minimally invasive pulmonary interventions. This roadmap aims to support interventional pulmonologists in the initial integration of CBCT into clinical practice.

## Figures and Tables

**Table 1:** Functionality comparison of mobile CBCT scanners.

Functionality	GE Healthcare OEC 3D	Siemens Cios Spin	Ziehm Vision RFD 3D
Field of View	19 cm × 19 cm × 19 cm	16 cm × 16 cm × 16 cm	16 cm × 16 cm × 16 cm 10 cm × 10 cm × 10 cm 19.6 cm × 19.8 cm × 18 cm
Volume Resolution	512	512	320 (standard) 512 (hi-res)
Scan Time	30 s	30/60 s	48 s
Reconstruction Time	30 s	30 s	8 s (standard) 18 s (hi-res)
Degrees of Scan for 3D	200°	196°	180°
Projections	200 or 400	100/200/400	200 or 400
Isocentric	Yes	Yes	Multi-movements
Augmented Fluoroscopy	Yes	No	No

**Table 2:** Cost impact of non-diagnostic navigational bronchoscopy (CBCT business case).

Parameter	Value	Notes
Study period	January 2023–December 2024	Retrospective service evaluation
Total procedures analysed	720	Navigational bronchoscopies
Initial diagnostic yield	82% (590/720)	First-attempt diagnosis
Non-diagnostic cases	130	Required further intervention
Repeat bronchoscopies	40	Malignancy confirmed in 35
Surgical cases requiring frozen section	95	Following non-diagnostic bronchoscopy
Mean frozen section time	80 min	Intraoperative waiting time
Average stapler cost per case	EUR 600	Approx. 3 cartridges (EUR 200 each)
Total additional cost (excluding OR time cost)	EUR 59,000	Includes consumables and sample transportation
Total additional operating room time	126 h	Compared to upfront definitive surgery

**Table 3:** Implementation pathway for CBCT-guided bronchoscopy.

Step	Phase	Core Objective	Key Actions
1	Infrastructure audit	Identify available resources	Locate CBCT scanner Assess compatibility
2	Entry strategy	Secure access	Negotiate access
3	Case preparation	Establish readiness	Training Logistics Ventilation Safety
4	Case selection	Ensure early success	Select low-risk, accessible lesions
5	Team building	Assemble expertise	Include experienced operators and support
6	Workflow familiarisation	Identify inefficiencies	Review early cases Refine processes
7	Scope expansion	Gradual scaling	Introduce more complex cases
8	Optimization	Improve efficiency	Standardize setup Reduce procedure time
9	Business case development	Justify investment	Analyse outcomes and costs
10	Program expansion	Increase adoption	Promote internally and externally

**Table 4:** Minimum requirements for establishing a CBCT-guided bronchoscopy program.

Domain	Minimum Requirements	Key Considerations
Imaging infrastructure	CBCT-capable scanner	Hybrid OR, IR, or dedicated suite
Bronchoscopy equipment	Flexible bronchoscope Biopsy tools	Compatibility with navigation systems
Fixation system	Bronchoscope holder	Must meet safety and sterility standards
Personnel	Interventional pulmonologist Anaesthesia team Radiology technologist	Multidisciplinary coordination essential
Anaesthesia support	Ventilation protocol	Atelectasis prevention and motion reduction
Imaging support	Fluoroscopy + Lung CBCT software	Augmented fluoroscopy beneficial
Safety protocols	Radiation protection Room-exit workflow Standardized room setup Procedural checklist	Staff exposure minimization
Workflow organisation	Standardized room setup Procedural checklist	Reduces variability
Training	Observership Proctoring Simulation	Shortens learning curve
Governance	Institutional approval Safety compliance	Especially for non-standard devices

## Abbreviations

The following abbreviations are used in this manuscript:

CBCT	Cone Beam Computed Tomography
IR	Interventional Radiology
OR	Operating Room
TiL	Tool-In-Lesion

## References

[1] Setser R., Chintalapani G., Bhadra K., Casal R.F. (2020). Cone beam CT imaging for bronchoscopy: A technical review. J. Thorac. Dis..

[2] Balasubramanian P., Abia-Trujillo D., Barrios-Ruiz A., Garza-Salas A., Koratala A., Chandra N.C., Yu Lee-Mateus A., Labarca G., Fernandez-Bussy S. (2024). Diagnostic yield and safety of diagnostic techniques for pulmonary lesions: Systematic review, meta-analysis and network meta-analysis. Eur. Respir. Rev..

[3] Verhoeven R.L.J., Kops S.E.P., Wijma I.N., Ter Woerds D.K.M., van der Heijden E.H.F.M. (2023). Cone-beam CT in lung biopsy: A clinical practice review on lessons learned and future perspectives. Ann. Transl. Med..

[4] Richmond H., Copsey B., Hall A.M., Davies D., Lamb S.E. (2017). A systematic review and meta-analysis of online versus alternative methods for training licensed health care professionals to deliver clinical interventions. BMC Med. Educ..

[5] Bhadra K., Setser R.M., Condra W., Pritchett M.A. (2022). Lung Navigation Ventilation Protocol to Optimize Biopsy of Peripheral Lung Lesions. J. Bronchol. Interv. Pulmonol..

[6] Pritchett M.A., Lau K., Skibo S., Phillips K.A., Bhadra K. (2021). Anesthesia considerations to reduce motion and atelectasis during advanced guided bronchoscopy. BMC Pulm. Med..

[7] Dai S., Xu G., Chen Z., Tang J. (2024). Intraprocedural computed tomography-guided navigation with ventilatory strategy for atelectasis (ICNVA): A modified electromagnetic navigation bronchoscopy. J. Thorac. Dis..

[8] Khan A., Bashour S.I., Casal R.F. (2023). Preventing atelectasis during bronchoscopy under general anesthesia. J. Thorac. Dis..

[9] Salahuddin M., Sarkiss M., Sagar A.S., Vlahos I., Chang C.H., Shah A., Sabath B.F., Lin J., Song J., Moon T. (2022). Ventilatory Strategy to Prevent Atelectasis During Bronchoscopy Under General Anesthesia: A Multicenter Randomized Controlled Trial (Ventilatory Strategy to Prevent Atelectasis—VESPA—Trial). Chest.

[10] Boster J.M., Goertzen M., Sarkiss M., Armas Villalba A.J., Bhandari B.S., Song J., Jimenez C.A., Sabath B.F., Lin J., Grosu H.B. (2026). Superiority of Lateral Decubitus Strategy in Preventing Atelectasis from Obscuring Targets During Robotic Bronchoscopy: Lateral Decubitus Strategy vs Ventilatory Strategy to Prevent Atelectasis Trial. Chest.

[11] Lin J., Sabath B.F., Sarkiss M., Jimenez C.A., Casal R.F. (2022). Lateral Decubitus Positioning for Mobile CT-guided Robotic Bronchoscopy: A Novel Technique to Prevent Atelectasis. J. Bronchol. Interv. Pulmonol..

[12] Kops S.E.P., Heus P., Korevaar D.A., Damen J.A.A., Idema D.L., Verhoeven R.L.J., Annema J.T., Hooft L., van der Heijden E.H.F.M. (2023). Diagnostic yield and safety of navigation bronchoscopy: A systematic review and meta-analysis. Lung Cancer.

[13] Bruinen A.R.C., Verhoeven R.L.J., Hannink G., van der Heijden E.H.F.M. (2026). Robotic versus CBCT navigation bronchoscopy: Propensity matched analysis of diagnostic yield. ERJ Open Res..

[14] Bruinen A.R.C., Verhoeven R.L.J., van der Heijden E.H.F.M. (2025). Cone beam computed tomography for navigational bronchoscopy. J. Thorac. Dis..

